# Prediction of Intrinsic Disorder in MERS-CoV/HCoV-EMC Supports a High Oral-Fecal Transmission

**DOI:** 10.1371/currents.outbreaks.22254b58675cdebc256dbe3c5aa6498b

**Published:** 2013-11-13

**Authors:** Gerard Kian-Meng Goh, A. Keith Dunker, Vladimir Uversky

**Affiliations:** Goh's BioComputing, Singapore, Singapore; Indiana University School of Medicine, Indianapolis, Indianna, USA; University of South Florida, Tampa, Florida, USA

## Abstract

A novel coronavirus, MERS-CoV (NCoV, HCoV-EMC/2012), originating from the Middle-East, has been discovered. Incoming data reveal that the virus is highly virulent to humans. A model that categorizes coronaviuses according to the hardness of their shells was developed before the discovery of MERS-CoV. Using protein intrinsic disorder prediction, coronaviruses were categorized into three groups that can be linked to the levels of oral-fecal and respiratory transmission regardless of genetic proximity. Using this model, MERS-CoV is placed into disorder group C, which consists of coronaviruses that have relatively hard inner and outer shells. The members of this group are likely to persist in the environment for a longer period of time and possess the highest oral-fecal components but relatively low respiratory transmission components. Oral-urine and saliva transmission are also highly possible since both require harder protective shells. Results show that disorder prediction can be used as a tool that suggests clues to look for in further epidemiological investigations.

## Introduction

**MERS-CoV/HCoV-EMC: A novel coronavirus that is highly virulent**

In June 2012, a 60-years old Saudi man was hospitalized with acute respiratory symptoms and was tested to be negative for diseases known at that time. He also suffered from renal failure and died later. Samples were sent to the Eramus Medical Center (EMC), Netherlands, and the virus isolated in sample was verified to be a coronavirus that was previously unknown. Therefore, this new virus was originally named HCoV-EMC, for human coronavirus – Eramus Medical Center.^1^^,^ 2 Although this name is used in most current publications and in UniProt, this virus is also known by other names, such as NCoV (novel coronavirus, an initial temporary name), and MERS-CoV (short for Middle East respiratory syndrome coronavirus, the latest terms approved by WHO on May 15, 2013, http://www.euro.who.int/en/what-we-do/health-topics/communicable-diseases/influenza/news/news/2013/05/novel-coronavirus-update-new-virus-to-be-called-mers-cov). In this manuscript, two names, MERS-CoV and HCoV-EMC, will be interchangeably used to refer this new virus.

The new coronavirus seems to be highly virulent. By April 2013, 11 of the 17 people that were known to be infected died. As of early May 2013, a total of 23 cases of the disease were detected in multiple countries (Saudi Arabia, Jordan, Germany, and he United Kingdom), with 16 death, and with the Saudi Arabian city of Riyadh having the majority of the deaths (11 deceased). As of May 15, WHO reported a global total of 40 laboratory-confirmed cases of human infection with MERS-CoV, including 20 deaths, in 6 countries: Saudi Arabia, France, Germany, Jordan, Qatar, and the United Kingdom. So far, those who were infected with MERS-CoV had either lived in or visited the Middle-East. Therefore, there are well-justified fears that the newly discovered virus could lead to an outbreak that would resemble the SARS-CoV (Severe Acute Respiratory Syndrome – Coronavirus) epidemic of 2002-4.^1^

HCoV-EMC has been, however, found to be quite different from SARS-CoV. For instance, it is currently known that MERS-CoV is not as infectious as SARS-CoV in terms of human to human transmission.^3^ On the other hand, MERS-CoV is more virulent than SARS-CoV. Infection with MERS-CoV, unlike SARS, often comes with multiple organ failures,^2^ including rapid kidney failure. The receptor responsible for viral-entry in the case of MERS-CoV has been found to be different from that of SARS-CoV. There are still many questions related to MERS-CoV/HCoV-EMC that need to be answered. We address some of the questions and attempt to provide some insights using predictions of protein intrinsic disorder content as a tool.

**Goals**

Our paper, “Understanding Viral Transmission Behaviors Using Protein Intrinsic Disorder”^4^ was written before the discovery of MERS-CoV. That paper described a model developed by measuring predicted disorder of two shells proteins of coronaviruses. The model grouped the analyzed viruses by the hardness of their shells. The categorization was found to be correlated with the various transmission modes. For example, the SARS-CoV was found to have moderate levels of disorder suggesting that it belonged to the category B, which explained the virus’s moderate ability to spread via respiratory mode but yet having sufficiently large “oral-fecal” component to last outside the physiological environment for a reasonably long period of time. The immediate question that then arises is: Where does MERS-CoV stand in this case? As a result of the recent availability of protein sequence data for this virus, we are able to report on its categorization and the potential implications of this categorization based on the analysis of the propensity of viral proteins for intrinsic disorder. Here, we applied the model that was developed and described in the previous paper^4^ to the MERS-CoV to provide a glimpse into the evolutionary nature of this virus using protein intrinsic disorder at its shell as a dissecting tool.

**Protein intrinsic disorder**

Abundantly found in various proteomes, intrinsically disordered proteins (IDPs) or hybrid proteins, comprising ordered and IDP regions (IDPRs), are biologically active proteins without unique 3D-structures.^5^^-^ 28 IDPs/IDPRs are characterized by a wide functional spectrum, being commonly involved in various signaling, regulation, and recognition processes.^5^^-^ 13 ^,^ 16 ^,^ 17 ^,^ 29 ^-^ 40. The functional diversity provided by intrinsic disorder in IDPs/IDPRs is complementary to the functions of ordered proteins and domains.^37^^-^ 39 Numerous IDPs/IDPRs are associated with various human diseases.^41^

Amino acid sequences encoding IDPs/IDPRs are different from sequences of structured globular proteins and their domains at several levels, including amino acid compositions, sequence complexity, hydrophobicity, charge, flexibility, etc. For example, in comparison with structured proteins and domains, IDPs/IDPRs are depleted in order-promoting residues W, C, F, Y, I, L, V, and N and are enriched in disorder-promoting amino acids A, R, G, Q, S, P, E, and K.^7^^,^ 42 ^-^ 45 Based on these sequence divergence, various computational tools have been developed and used to detect the presence and characterize the distribution peculiarities of disorder in proteins and proteomes. One of these tools is PONDR^®^ -VLXT,^46^^,^ 47 which is a neural network that uses protein sequence as its input. This predictor of protein intrinsic disorder has been used to identify several trends and peculiarities pertaining to many viruses in general,^26^ and also to some specific viruses, such as HPV,^48^ HIV,^49^^-^ 51 SARS-CoV,^4^ and influenza A H1N1 1918.^52^

## Methods

The protein intrinsic disorder-based model for the classification of coronaviruses was developed before the discovery of MERS-CoV. A full description of this model can be found in our previous paper.^4^ The model basically measures the percentage of intrinsic disorder (PID) in the two major coronavirus shell proteins, the M and N proteins. The PID is defined as the number of protein residues that are predicted to be disordered divided by the total number of residues.^49^

The model was based on the assumption that the viral shells protect the virion from damage in the physiological and non-physiological environments.^50^ Based on this reasoning, the model divided coronaviruses by the hardness of their shell and, as a result, was also able to categorize coronaviruses into three groups that have varying levels of oral-fecal and respiratory transmission.^4^ In the previous paper, MANOVA was used to show that the model is able to classify the various viruses into identifiable groups. The same analysis, was re-used to confirm that MERS-CoV and bat CoV-HKU4/5 fall within the framework of the model already developed.^4^

The new set of data was entered using JAVA™-JDBC programs developed previously. The database used in the analysis has been previously described.^49^ The JAVA program that could generate codes readable in Jmol was reused to generated three dimensional figures annotated by disorder prediction.^50^^,^ 52 ^,^ 53 Data from Protein Data Bank (PDB, http://www.ncbi.nlm.nih.gov/Structure/index.shtml)^54^ and UniProt (www.UniProt.org)^55^ were downloaded using JAVA programs.

## Results

**Categorizing MERS-CoV: Disorder group C**

The model proposed in our previous paper^4^ was able to classify coronaviruses on the basis of disorder level (PID, percentage of intrinsic disorder) in the shell proteins; i.e., the M- and N-protein as seen in Table 1. The breakdown resulted in an automatic categorization of the viruses by transmission behaviors, irrespective of the genetic and antigenic proximities of the analyzed viruses.^4^ The three groups (disorder groups A, B and C) arose from this analysis.

**Table 1. Categorization of MERS-CoV among coronaviruses by disorder groups.** Based on the rule previously developed, MERS-CoV has be categorized as part of disorder group C. Statistical analysis using the additional data yields even higher significance (MANOVA, p < 0.001, F=17.96).

Disorder Group	Coronavirus	PID in M-proteins (UniProt ID)	PID in N-proteins (UniProt ID)	Remarks
A	IBV(Avian) HCoV-229E	9.8(P69606) 23(P15422)	56(Q8JMI6) 56(P15130P	Most Disordered Group. Higher Respiratory Transmission. IBV M-protein is quite ordered.
B	SARS-CoV PEDV Bovine Canine(Resp.) HCoV-OC43 HCoV-NL63 Bat* Bat-HKU4 Bat-HKU5	8(P59596) 8(P59771) 7.4(P69704) 6.5(A3EXD6) 7(Q4VID2) 11(Q6Q1R9) 11.5(A3EXD6) 16(A3EXA0) 11(A3EXD6)	50(P59595) 51(Q07499) 53(Q8V432) 51(A3E2F7) 51(P33469) 49(Q6Q1R8) 47(Q3LZX4) 48(A3EXA1) 47(A3EXD7)	Moderately Disordered Group. Intermediate levels of fecal-oral/contact transmission. Moderate levels of respiratory transmission. Bat-CoVs are placed in the group B because although Bat-HKU4 and HKU5 have somewhat lower nucleocapsid (N) PIDs, they have higher matrix (M) PIDs.
C	MHV TGEV **MERS-CoV**Canine(Ent.) HCoV-HKU1	8(Q9JEB4) 14(P09175) **9(K0BU37)** 8(B8RIR2) 4(Q14EA7)	46(Po3416) 43(P04134) **44(K0BVN3)** 40(Q04700) 37(Q0ZME3)	Least disordered group. Higher levels of fecal-oral and contact transmission, lower respiratory transmission.

In this protein intrinsic disorder-based classification, the most disordered group A included coronaviruses that had the highest respiratory transmission and the least oral-fecal transmission. In this group, however, the avian IBV (Infectious Bronchitis Virus) had a rather markedly hard outer shell, which is likely a sign of the ability of this virus to remain in fecal masses or urine for a relatively longer period of time than viruses with less rigid shells. Moderately disordered group B, that included SARS-CoV and PEDV (Porcine Epidemic Diarrhea Virus), had moderate levels of both respiratory and oral-fecal transmission. The least disordered group C consisted of viruses that had higher oral-fecal transmission but possessed lower respiratory transmission.

**MERS-CoV falls squarely into the group C**

As discussed in the case of IBV, not all viruses that have high respiratory transmission in group A, have also soft (more disordered) outer shells. A hard outer shell in group A could mean higher fecal-respiratory or urine-respiratory potential. Similarly, not all viruses in group C have hard outer shells, especially if the virus spreads rapidly without the necessity of having to be left in the environment for a long time, which is the case of TGEV. The availability of MERS-CoV genetic and protein sequence information made it possible to evaluate the PID levels of its M- and N-proteins. The results of this analysis are shown in Table 1, which suggests that MERS-CoV falls into the disorder group C, which includes coronaviruses with higher oral-fecal component. Although according to the disorder criteria, MERS-CoV is in the same category as TGEV, unlike TGEV, it has a rigid M-protein that resembles the M-proteins of IBV and SARS-CoV.

**MERS-CoV: Hard shells**

Figure 1 shows that the TGEV and MERS-CoV are rather similar. There are, however, marked differences in the amount of disorder found in their M-proteins. This is likely an indication of a greater persistence of MERS-CoV in the environment defined by the relative rigidity of both of its shell proteins. Figure 1 also illustrates the fundamental differences between the members of the various groups and clearly shows that the highest levels of disorder can be found in the group A, especially in the N-proteins of this group.

**Graphical comparison of the PID in MERS-CoV and other coronaviruses.**
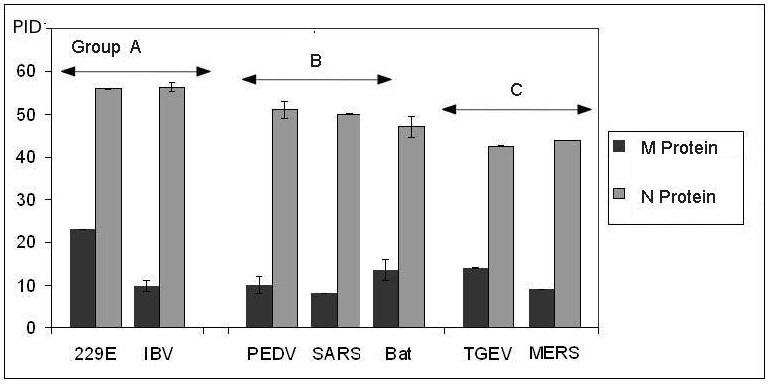
The chart illustrates the pair-wise similarity of disorder contents in M- and N-proteins of MERS-CoV and TGEV belonging to the category C. M- and N-proteins of SARS-CoV and PEDV (both are from the category B) also possess similar PID contents, whereas the proteins of the bat coronavirus are located at the borderline between the categories B and C. Group A (HCoV 229E and IBV) is characterized by the most disordered M- and N-proteins.

**Bat HKU4/5 vs. MERS-CoV**

The bat coronavirus species used in this study (HKU4 and HKU5)^56^ are more closely related to MERS-CoV than viruses used in the previous publication.^4^ A comparison of the PID of the bat CoV species used here and the bat CoV samples in the previous paper shows little differences especially for the N-protein even though slight differences in the M-protein are seen. Other bat samples show larger differences only for the M-protein. The fact that the disorder content in N-proteins is conserved is is quite surprising since bat coronaviruses are known to be genetically diverse.^1^^,^ 56 ^,^ 57 Also, it should be noted that in Figure 1 and Table 1, bat coronaviruses are placed in disorder group B since the PID of the N-protein is slightly lower compared to that of the SARS-CoV but the M-protein PID tends to be higher than usual, even though this is not always the case for all bat CoV. Therefore, bat coronaviruses, at least for the samples we analyzed here, should remain in group B but, perhaps, at the boundary between the groups B and C, as seen in Figure 1.

**Higher statistical significance in grouping using additional data from MERS-CoV and bat HKU4/5-CoV**

As mentioned in Table 1, the addition of data pertaining to MERS-CoV and bat-HKU4/5 provides us with an even higher level of statistical significance (MANOVA, p<0.0001, F=17.96) when compared to the analysis of a smaller number of viruses done in our previous paper (p<0.05, F=16.3). This basically means that the categorization of the additional items into their corresponding disorder group is within the rules we have previously developed for the identification of a virus group and also reaffirms that the groups remain identifiable.

Among the protein used in our study (see Table 1), of particular interest is the new set of bat coronaviruses, analysis of which will allow us to see the differences in the shells despite their genetic closeness. Murine hepatitis virus (MHV) is included in this study since the structure of its N-protein is the only structure of the nucleocapsid protein of the coronaviruses in disorder group C that is currently available in the PDB. While MHV is categorized to be in disorder group C (since the M-protein is relatively hard for, at least, certain strains) the disorder level of its N-protein is not too dissimilar to that of the bat coronaviruses suggesting that MHV is likely to be near the boundary between groups B and C.

**Qualitative *vs.* quantitative data: Use of per-residue disorder predictors and three dimensional structures**

Results of disorder evaluation in some of the M- and N-proteins from the coronaviruses listed in Table 1 are shown in Figure 2 and 3. For this analysis, the PONDR-FIT algorithm,^58^ a computational tool representing a meta-predictor that combines six individual predictors, which are PONDR^®^ VLXT,^42^ PONDR^®^ VSL2,^59^ PONDR^®^ VL3,^60^ FoldIndex,^61^ IUPred,^62^ and TopIDP,^63^ was used. This meta-predictor is moderately more accurate than each of the component predictors and provides accurate disorder predictions at the residue level. The residue-level predictions allow for a more insightful analysis, including an investigation into the number and size of the predicted disordered segments. Figure 2 shows that the M-proteins from IBV, SARS-CoV, MHV, Bat-HKU4, Bat-HKU5, and MERS-CoV are predicted to be mostly ordered, possessing disordered termini. Curiously, the disorder profiles of the M-proteins from Bat-HKU4 and MERS-CoV are rather similar and the two disorder profiles have many common features (e.g., pronounced disorder spikes around residues 38 and 135 and rather similar sets of peaks in the vicinity of residues 75 and 140).

**Disorder propensities of the coronaviral M-proteins.**
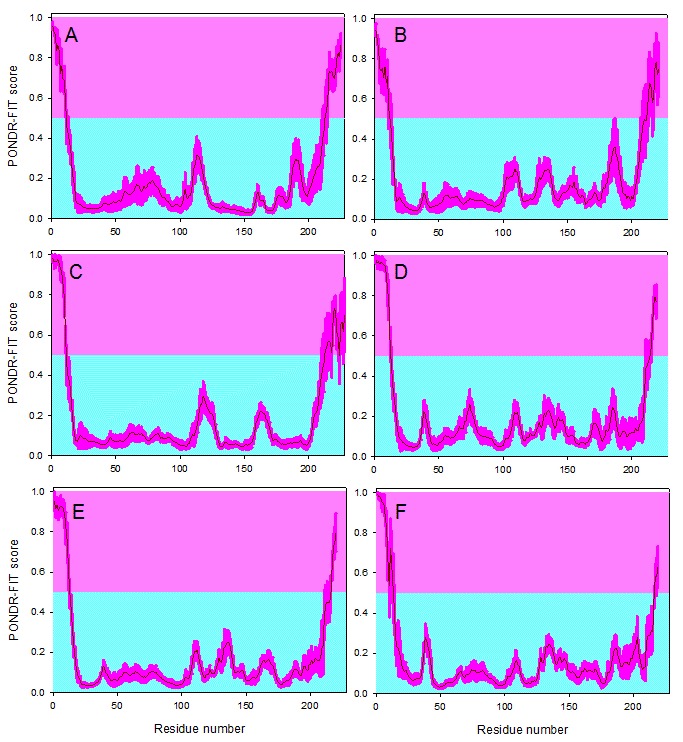
Evaluating disorder propensities of the M-proteins from IBV (A), SARS-CoV (B), MHV (C), Bat-HKU4 (D), Bat-HKU5 (E), and MERS-CoV (F) by the PONDR-FIT algorithm. Scores above 0.5 correspond to the disordered residues/regions (shaded in light pink), whereas scores below 0.5 indicate residues/regions predicted to be ordered (shaded in light cyan). Pink shades around the PONDR-FIT curves (dark red) reflect the distributions of errors in evaluating the disorder scores.

Figure 3 illustrates that the analyzed coronaviral N-proteins are predicted to possess significant amount of intrinsic disorder in a form of relatively long disordered regions. It is clear, however, that these proteins should be classified as hybrid proteins, since they contain both ordered domains and disordered regions. Comparison of disorder profiles calculated for different N-proteins shows that the MERS-CoV protein looks as a hybrid between the IBV and Bat-HKU5 proteins. In fact, there is a close similarity between the N-terminal halves of the IBV and MERS-CoV N-proteins, whereas the C-terminal domain of the MERS-CoV N-protein is strikingly similar to the C-terminal domain of the Bat-HKU5 N-protein. The fact that significant parts of the M- and N-proteins of MERS-CoV are rather similar to those of bat-CoVs suggests that MERS-CoV may originate from bat coronavisures.

**Disorder propensities of the coronaviral N-proteins.**
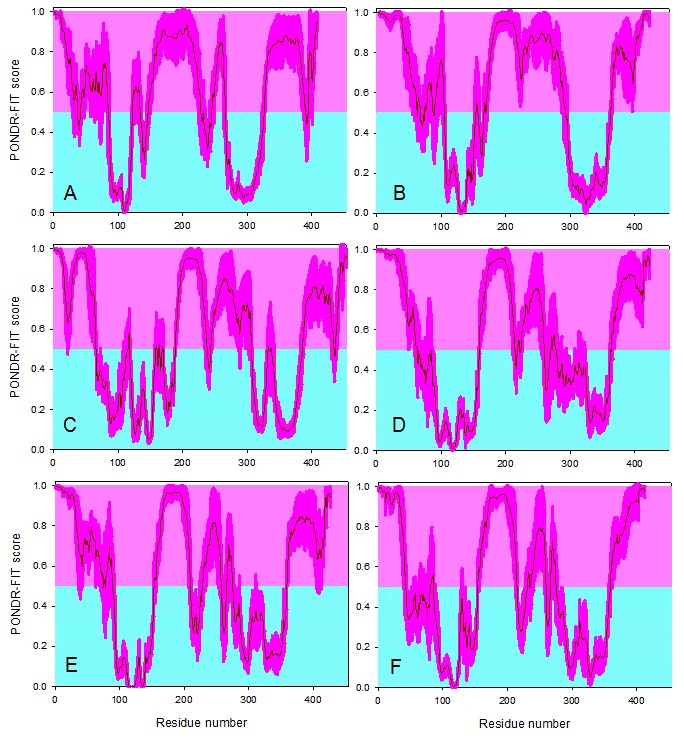
Analysis of the disorder propensities of the N-proteins from IBV (A), SARS-CoV (B), MHV (C), Bat-HKU4 (D), Bat-HKU5 (E), and MERS-CoV (F) by the PONDR-FIT algorithm. Scores above 0.5 correspond to the disordered residues/regions (shaded in light pink), whereas scores below 0.5 indicate residues/regions predicted to be ordered (shaded in light cyan). Pink shades around the PONDR-FIT curves (dark red) reflect the distributions of errors in evaluating the disorder scores.

Figure 4 represents 3D-structures of the ordered domains of N-proteins of three coronaviruses from different disorder groups, IBV (group A), SARS-CoV (group B), and MHV (group C). Structural information is available for the RNA-binding domains of the N-proteins from all three viral species considered here (PDB codes 2BXX, 1SSK, and 3HD4, for nucleoproteins from IBV, SARS-CoV, and MHV, respectively), as well as for the dimerization domains of the nucleoproteins from IBV and SARS-CoV (PDB codes 2CA1 and 2JW8, respectively).

**Structures of the ordered domains of the N-proteins.**
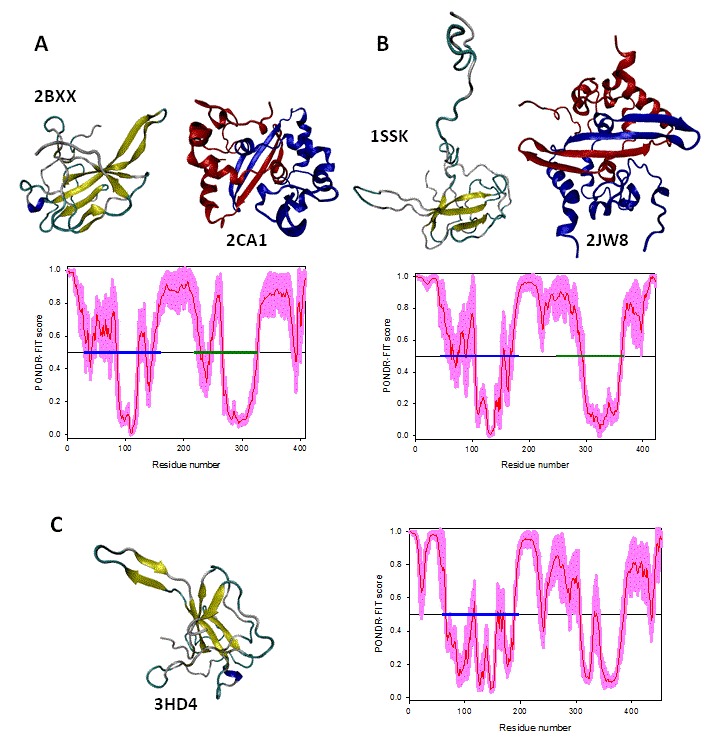
3D-structures of the ordered domains of N-proteins of three coronaviruses from different disorder groups, group A: IBV (A), group B: SARS-CoV (B), and group C: MHV (C). In addition to the available 3D-structures of the RNA-binding and dimerization domains, each panel contains the PONDR-FIT profile, where the localization of these domains is shown by blue and dark yellow bars, respectively. For IBV, the shown structures of the RNA-binding and dimerization domains are 2BXX (residues 29-160) and 2CA1 (residues 218-326). For SARS-CoV, structures of the RNA-binding and dimerization domains are 1SSK (residues 45-181) and 2JW8 (residues 248-365). For MHV, the only available structure is the structure of the RNA-binding domain (PDB code: 3HD4, residues 60-197). Structures were drawn using the VMD software.^83^

Structural alignments for these domains are shown in Figure 5. Here, Figure 5A represents structurally aligned RNA binding domains of the nucleoproteins from IBV (blue), SARS-CoV (red), and MHV (grey), whereas structurally aligned oligomerization domains of the N-proteins from IBV (blue) and SARS-CoV (red) are shown in Figure 5B. Figure 5 clearly shows that despite sequence divergence, the ordered domains of these nucleoproteins are structurally rather similar.

**Structural alignments of the ordered domains of coronaviral N-proteins.**
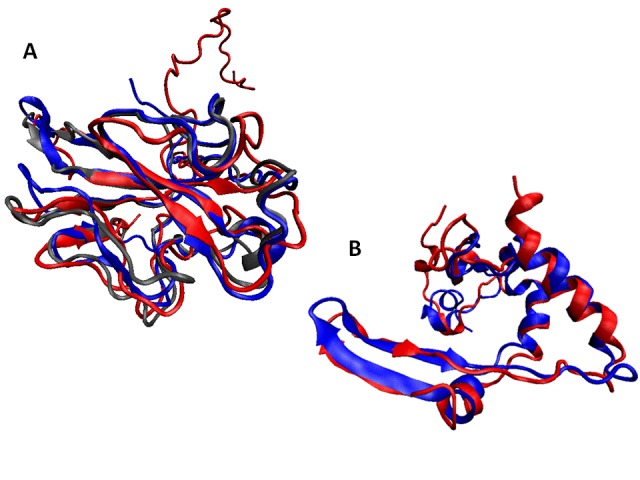
Structural alignments of the RNA-binding domains (A) and dimerization domains (B) of the N-proteins from three representative members of the coronavirus family, IBV (blue structures), SARS-CoV (red structures) and MHV (grey structure). The structural alignment was done by MultiProt (http://bioinfo3d.cs.tau.ac.il/MultiProt/).^84^ The aligned structures were drawn using the VMD software.^83^

## Discussion

**A glimpse into the virus evolutionary past using protein intrinsic disorder prediction**

Incoming data pertaining to the MERS-CoV present an intriguing and somewhat puzzling picture of the virus. The virus is apparently not easily transmissible between humans especially when compared to SARS-CoV.^3^^,^ 64 When SAR-CoV struck in 2002-4, a sizable number of people fell ill when they were in proximity of infected individuals,^65^ and a large number of healthcare workers also fell ill when they treated patients with SARS.^65^ This behavior has not been observed so far for MERS-CoV. However, although healthcare workers were not originally infected by this virus,^3^ a recent WHO report notified the existence of the alarming possibility for health-care-associated transmission of the virus, since two health care workers were diagnosed with MERS-CoV infection after exposure to patients (http://www.euro.who.int/en/what-we-do/health-topics/communicable-diseases/influenza/news/news/2013/05/novel-coronavirus-update-new-virus-to-be-called-mers-cov).

There is, however, enigmatic evidence coming from the laboratory. MERS-CoV was found to bind to a different receptor with respect to SARS-CoV. MERS-CoV binds to the di-peptidyl peptidase-4 (DPP4), whereas SARS-CoV has affinity for angiotensin-converting enzyme 2 (ACE2).^2^^,^ 66 ^,^ 67. MERS-CoV has experimentally been shown to be highly adapted to the cells of a large variety of animals, even more than SARS-CoV.^2^^,^ 66 ^,^ 68 Furthermore, there is evidence that MERS-CoV spread rapidly in human epithelial cells at a pace that is faster than that of SARS-CoV.^1^^,^ 68 How could a virus be less transmissible when experimental results show that it is highly adapted to human cells? We shall see later that the protein intrinsic disorder-based model can account for this apparent paradox.

A way that scientists attempt to answer the question of infectivity of MERS-CoV is by looking at the locations and distribution of the receptors in lungs.^2^ Such approach is not without difficulty for two reasons. Firstly, at the moment very little is known about the distribution of DPP4 in the lungs.^66^ Secondly, little attention has been paid to the affinities of the viruses to the receptors. An entirely different approach has been described in our previous paper.^4^ It involves examining the hardness of the viral shells. The hardness/rigidity of viral shells can tell us much about the modes of transmission if we assume that a major role of the shell proteins is to protect the virion from damage by physiological or non-physiological environments.

**Predicted higher oral-fecal transmission explains virus behavior thus far**

We have seen that our model places MERS-CoV in disorder group C, which comprises coronaviruses with lower levels of intrinsic disorder in their M- and N-proteins that translates into higher levels of potential oral-fecal transmission. This group includes TGEV and HKU1 (Table 1 and Figure 1). The viruses in group C are distinct from SARS-CoV, which falls into group B. SARS-CoV is thus analyzed as having moderate oral-fecal and respiratory transmission. Signs of higher respiratory transmission were detected by the model in coronaviruses such as IBV and HCoV-229E (group A), but such higher levels of respiratory transmission could not be detected in SARS-CoV, and, even less so, in MERS-CoV. The identifiable sign of higher respiratory transmission is the higher level of predicted intrinsic disorder especially in the coronaviral N-proteins.

Is the expectation of a higher oral-fecal potential for MERS-CoV supported by incoming data thus far? This hypothesis is not only supported but it also explains much of the perplexing data pertaining to MERS-CoV. For example, it was not clear why the SARS-CoV spread relatively easily when someone is infected, whereas, in the case of MERS-CoV, fewer people in contact with the infected individuals became infected themselves. Our model explains that the evolution of MERS-CoV is such that it facilitates a larger oral-fecal transmission potential for its survival, but SARS-CoV, on the other hand, needs a larger respiratory transmission level and lower oral-fecal transmission to be successful, with the respiratory and oral-fecal routs of the SARS-CoV transmission still being relatively moderate. We will see that the higher oral-fecal potential of MERS-CoV explains much of the underlying reasons for its virulence, its high adaptability to a broad range of animal hosts, and its apparent lack (or at least highly diminished efficiency) of human-human transmission in comparison with SARS-CoV.

Higher oral-fecal provides a good mean for spread among many kinds of animals

As described in our previous paper, our model suggested that the reason why PEDV spreads less easily in pigs than TGEV is because PEDV possesses lower oral-fecal transmission that hinders the rapid spread among pigs.^4^ The higher oral-fecal potential, as suggested by the lower PID scores for TGEV N-protein,^4^ provides a crucial means for the rapid spread among pigs. As seen in Table 1 and Figure 1, while PEDV resembles more SARS-CoV in its shell disorder (PID: 51% for N and 8% for M in PEDV vs. 50% for N and 8% for M in SARS-CoV), TGEV (PID: 43% for N and 14% for M) resembles MERS-CoV (PID: 43% for N and 9% for M) especially with regard to the amount of disorder in their N-proteins. This similarity gives some hints on the behavior of MERS-CoV, where this virus was able to infect animals possessing behaviors that confer advantage to the virus in its oral-fecal spread, and the virus was thus able to spread rapidly. It should be reminded that higher oral-fecal transmission means higher chances of infection via close contacts and touch.^69^ The inherent behaviors of a wide variety of animals are likely to confer larger advantages to such viruses with larger oral-fecal transmission. We will see later that this factor has implications for the evolution of virus in acquisition of its adaptability to cells of a wide variety of animals.

Our model suggests that higher respiratory potential and moderately hard shell allows the virus to persist in the environment where it is able to infect via respiratory means, including having greater chances of infection via respiratory-fecal mode. In agreement with this hypothesis, it has been observed that the unexposed herds of swine can be infected by PEDV without direct contact with infected pigs by simply moving pens that were occupied by infected pigs, even if the pen was cleansed. This possibility was also the case for SARS-CoV transmission when SARS patients could not recall having met those who infected them. TGEV, like other viruses in disorder group C, rely on close contact, touch, and oral-fecal routes.^4^^,^ 69 This also explains the currently observed behavior of MERS-CoV, which is not seen as easily transmissible from humans to humans even though it is known to infect human cell-lines easily.

While SARS-CoV, PEDV, and IBV are likely to have higher fecal-respiratory potential than TGEV has, it would, however, be a gross misinterpretation of the analysis above to conclude that fecal-respiratory transmission is negligible or non-existent in MERS-CoV. There are many factors that we have yet to mention. Firstly, we need to bear in mind that even TGEV is known to have some fecal-respiratory potential.^70^^,^ 71 There is however, a difference with regard to MERS-CoV: It has a harder outer shell (PID of 9% in M-protein) than that of TEGV (M-protein has PID of 14%). This means that HCoV-EMC is more likely to remain intact in a non-physiological environment for a longer period of time, which gives it greater “shelf life” to infect its host by respiratory means or other routs, even if its respiratory transmission potential is lower. The amount of viral shedding also matters, and if MERS-CoV has a high oral-fecal component, it is, in theory, more likely to be shed in larger quantity as fecal matter or in urine.

**Oral-saliva and oral-urine routes are also a possibility for viral transmission**

The MERS-CoV has hard shells and ability to persist outside for a long time

It was noted that both PEDV and SARS-CoV are quite persistent outside the host.^70^^,^ 72 ^,^ 73 This characteristics has not been just observed clinically but has also been detected by the disorder model when these viruses were categorized as group B,^4^ which has moderate respiratory and oral-fecal transmission. However, as mentioned above, disorder predictins were able to detect a relatively softer outer shell for TGEV (PID of 14% for the M-protein) when compared to shells of SARS-CoV and PEDV, the M-proteins of which both have PIDs of 8%.^4^ This seems like a contradiction: Why would a virus such as TGEV that has higher oral-fecal route have a softer shell? The answer is that apparently TGEV does not need to survive in the external environment for long since it spreads very rapidly among pigs^4 ^and having a more disordered shells has been known to offer viruses ways to evade the immune systems.^50^ This softness seen in the M-protein of TGEV could not be detected in the MERS-CoV, which has a PID of 9%. As mentioned, the model is apparently telling us that MERS-CoV has the ability to stay outside of the physiological environment for a longer period just like SARS-CoV or, perhaps, longer since we have not accounted for its more rigid N-protein (PID of 44% vs. 50% for MERS-CoV and SARS-CoV) that also plays a role in protecting the virion.

If MERS-CoV has a tendency for oral-fecal transmission, then why are patients infected with the MERS-CoV not showing signs of diarrhea, unlike many of the patients infected by SARS-CoV? A clue to the answer may lie in the observation that renal failure is common among MERS-CoV infected patients. Obviously, this is likely a clinical sign of the virus’s ability to invade the renal-urinary tract and may be a pointing hint to the possibility of oral-urine mode of transmission. Given the hard encasement of MERS-CoV detected by the model, there is no reason to dismiss this as a possibility either as a major or minor route of transmission since oral-urine transmission often entails the virus to remain in the external environment for extended period. Besides, virus has to enter the gastro-intestinal regions, where it is exposed to digestive acid and enzymes, before reaching the renal-urinary tract.

An interesting and related note is the fact that the other known coronavirus that causes renal-failure is avian IBV. The puzzling fact here is that the model has put IBV to be in group A, which is listed as having highest preference for respiratory transmission alongside HCoV-229E. However, IBV, unlike 229E, has a hard outer shell with its M-protein possessing PID of 9.8%, which is comparable to that of MERS-CoV (9.1%) even though IBV has a highly disordered inner shell (its N-protein has a PID of 56%) just like HCoV-229E. This may signify the presence of high urine-respiratory transmission in the case of IBV. The similarity in the PID scores for IBV and MERS-CoV M-proteins could reflect the need of both viral particles to remain in the environment for at least a reasonable period of time. It should also be kept in mind that while oral-fecal mode is an important mean for a virus to spread rapidly in many animals such as swine, higher respiratory transmission may be crucial for a virus to be highly infectious in birds as seen not just in IBV but also in viruses such as influenza A H5N1. There may also be advantages in transmission via urine by flying animals such as birds and bats since the fluid released during flight could provide wider contamination of areas with vegetations, possible nesting areas and edible fruits.

Just as we have seen that there are reasons to look into the possibility of oral-urine spread for MERS-CoV, there are also reasons to look into the possibility of MERS-CoV to be transmitted via saliva. Many viruses that have known association with oral-fecal transmission are also known to spread by oral-saliva routs (e.g., hantavirus and Nipah virus). In the case of Nipah virus, humans get infected after consuming raw fruits that were previously eaten by infected bats. Since MERS-CoV has the ability to infect bats and since of its close relatives, bat HKU4 and HKU5, are viruses infecting bats and flying foxes, the oral-saliva route needs to be scrutinized. Several factors that have been detected by the model allow us to believe that the transmission of MERS-CoV by saliva is quite possible. The first has to do with the fact that MERS-CoV is categorized into disorder group C. Another involves the prediction that its outer shell is hard. Related to these, it has been found that viruses that are in close contact with saliva are usually accompanied by a hard rigid shell as previously detected by disorder predictors, e.g. rabies (unpublished data from database mentioned in previous papers)^50^ and EIAV.^49^^,^ 50 These observations are consistent with the biochemistry of saliva since salivary enzymes, that include glycosidases and proteases, can damage the surface proteins and shells proteins found in the virion surface.^74^ Therefore, a hard shell is necessary to protect the virion from such damage especially if the virus has to remain in the saliva for a longer period.

One should keep in mind that although the proposed classification of coronaviruses into three groups based on the PID levels in their their N- and M-proteins is reliable and meaningful, it is unlikely that the PID criterion alone allows one to decisively conclude that MERS-CoV can be transmitted by saliva and/or urine. Therefore, the discussed above possibilities for such types of transmission are purely hypothetical.

**Need to look at other non-related viruses with parallel evolutionary experiences for clues to the behavior of a virus**

Looking at the evolutionary similar viruses can offer important clues

There is a tendency for scientists to overlook unrelated viruses that are evolutionary parallel or close to the virus of interest in their attempts to uncover clues.^75^ There are several possible reasons for this. For example, it is likely that many researchers may be confused by the concept that viruses that are evolutionarily similar are not necessarily genetically close or similar. Another reason is that little is yet known about the virus especially if the virus of interest is new. The protein intrinsic disorder model that was developed in our studies could play a role by providing initial evolutionary snapshots of a new virus that could be followed by searches for other viruses that may be evolutionarily similar and thereby, uncovering possible clues to its behaviors.

Many viruses that spread via oral-fecal or oral-urine mode are highly virulent especially to humans

A noticeable clinical characteristic of MERS-CoV is its high virulence with a mortality rate that far surpasses that of SARS-CoV.^3^ The mortality rate of patients infected with MERS-CoV is around 50-70%, compared to the 10-20% in case of the SARS epidemic and 3-10% in the case of the 1918 influenza pandemic.^2^ Given that the model is detecting a high oral/fecal/urine component, we need to be reminded that many viruses with a large oral/fecal/urine transmission potential can often inflict severe diseases with high mortality rates comparable to that of MERS-CoV. Examples of such include the Nipah virus,^76^ certain hantaviruses^77^ and Ebola virus.^78^ Like MERS-CoV, many of these viruses are highly adapted to cells of a wide variety of animals including human.^2^^,^ 66 ^,^ 76 ^,^ 77 ^,^ 79

Reason for virulence: High oral-fecal component may act as a barrier to the acquisition of herd immunity in some animals

It is quite possible that many of the viruses acquired pathogenesis as a result of oral-fecal route acting as a barrier that help rapid widespread infection among*some*species of animals and thus preventing the herd immunity that we see in case of some influenza viruses (except the current H5N1, which has a different barrier) and SARS-CoV. Over the course of the evolution, such viruses either remain virulent or acquire greater virulence since some animals species are unable to acquire immunity against viruses their immune systems have rarely seen.

The oral-fecal route is highly advantageous among a wide variety of animals: Evolutionary reason for promiscuous adaptability to a wide range of animal hosts

On the other hand, the same viruses may be able to adapt to a variety of animals since the oral-fecal transmission route is inherently favorable to infect many other types of animals. TGEV^80^ and Nipah virus^76^ are examples of viruses that spread rapidly among porcine herds. Nipah virus is highly virulent to humans but spreads rapidly among pigs often with only mild symptoms even though some do fall ill. Like MERS-CoV, it is also highly adapted to cells of a wide variety of animals including human. It is not hard to find other viruses that have adapted to a broad range of animal hosts and have large oral-fecal components. They include the Ebola virus and some species of hantaviruses.^77^^,^ 79

A glimpse into MERS-CoV’s evolution from bats using disorder prediction

While bats are yet to be proven to be the primary reservoir of MERS-CoV, there is some suspicion that MERS-CoV is closely related to the bat coronaviruses, HKU4 and HKU5.^56^ We should also keep in mind that spread by urine is yet to be shown as transmission route. It should be noted, however, that urine, saliva, and fecal spread by flying animals is common. Bats and flying foxes are known to spread virus-laden urine on wide area with edible fruits and vegetations that are accessible to a broad range of animals that feed on them and thus allowing the virus to enter into and adapt to a wide variety of animals. This can be seen, for instance, in Nipah virus.^76^ With this in mind, the model has much to say on how MERS-CoV could have arrived from the bat coronavirus relatives.

MERS-CoV may have acquired greater oral-fecal potential when it evolved from a virus meant solely for bats

We have seen in Figure 1 and Table 1 that bat coronaviruses belong to group B, just like SARS-CoV and PEDV. A closer look at the levels of disorder shows that they have somewhat more ordered N-proteins (47.5±1) when compared to, say, SARS-CoV (50%). The species analyzed here are HKU4 and HKU5, which are closely related to MERS-CoV.^56^ It should be noted that based on our small dataset, we can see that other unrelated bat coronaviruses have similar disorder levels in the N-proteins but the disorder variance for their M-proteins is high. An interpretation of this is that bat coronaviruses have the best fitness when they have a specific level of respiratory and oral-fecal transmission as dictated by the inherent nature of bats, but the viruses may vary in their ability to survive outside as seen by the varying hardness of the outer shell reflected in the disorder of their M-proteins. Interestingly, the level of disorder for the shell proteins of MERS-CoV is lower than that of HKU4/5, especially for the N-protein. The data is basically telling us that when the virus moved to adapt to a wider variety of animals it evolved by acquiring an even higher oral-fecal transmission capability with a harder shell so as to be able to last in the environment for a longer time.

Bat-CoV and MHV likely to lie at the borderline between groups B and C

Curiously, the protein intrinsic disorder data for bat CoVs show that despite their high genetic diversity, the PID scores for the N-proteins of these coronaviruses seem to cluster around 47%, while the disorder scores for their M-proteins vary in a wide range. This peculiarity suggests that bat-CoV are likely to be located at the borderline between disorder groups B and C. Strangely, the PID of the MHV N-protein (Figure 1 and Table 1) is also close to the disorder scores of the N-proteins of bats, thereby placing this virus at the borderline between groups B and C too. Given that both animals (mice and bats) often serve as natural reservoirs of many important viruses,^56^^,^ 76 ^,^ 81 a plausible hypothesis suggests that being at the borderline confers to the virus a larger flexibility in its future evolution and helps it to accommodate to other animal hosts.

## Conclusions

A higher level of oral-fecal transmission component was detected using protein intrinsic disorder predictions

A higher level of oral-fecal transmission is predicted for MERS-CoV, based on intrinsic disorder analysis of its shell, which positions it in disorder group C, which is the group that has the highest level of oral-fecal transmission and the relatively lowest levels of respiratory transmission. This is in sharp contrast to SARS-CoV and IBV, which are categorized as groups B and A respectively.

**Model is able to explain incoming data**

Incoming clinical and epidemiological data suggest that MERS-CoV is highly virulent, and is currently seen as not being highly transmissible among humans. On the other hand, experimental data show that the virus is highly adapted to cell-lines from a diverse range of animals including humans. By detecting a higher oral-fecal levels of transmission, the model is able to link the data coming from various sources. While having an oral-fecal transmission mode may have allowed the virus to enter into and move rapidly among a large number of animal species and thereby, acquiring its adaptability, it can also become or remain highly virulent in some species since the oral-fecal route acts as a natural barrier to quick transmission in those species. Evidence of this hypothesis can be seen in commonness of high virulence found in many viral diseases that rely largely on oral-fecal transmission for spread.

**Oral-fecal transmission and a lesser chance of a SARS-like epidemic**

A pressing question is: Does the larger levels of oral-fecal tranmission of MERS-CoV imply a lower chance of a SARS-like outbreak? Given the high pathogenicity of the virus, such an extrapolation must be made at the individual researcher’s own risk and should not be used as an excuse to let our vigilance down against the possibility of a SARS-like outbreak. There are several complex reasons for this. In order to make predictions with full confidence, one needs to assume that the model used has incorporated entity’s entire past. Unfortunately, such completeness is impossible in any model and the best that a model can offer is to provide a glimpse of an entity’s past, in this case, the evolutionary past of MERS-CoV. Furthermore, it should be reminded that many coronaviruses in disorder group C still have adequate respiratory components to be, at least, troublesome. An example is HKU1, which is known to spread widely among children since children are in environments where contacts and touch assist the transmission.^82^ Lastly, many viruses, such as Nipah virus, hantaviruses, and Ebola virus,^76^^,^ 77 that rely heavily on the oral/fecal/urine route are capable of repeated comebacks over decades in terms of causing outbreaks in humans, since, like MERS-CoV, they are highly adapted, and many have no lack of potential animal reservoirs, even if they are temporary. Over time, the death tolls arising from the repeated outbreaks may exceed that of a pandemic or epidemic. Obviously, there is no way for anyone to be certain if this will be the case for MERS-CoV, but it is a possibility to look out for.

**Model provides a glimpse into the virus evolutionary past: Useful epidemiological tool**

A model that provides us with a glimpse into the evolutionary past of a virus is likely to better serve as an epidemiological tool that could allow researchers to look into areas that would have been otherwise missed. More specifically, as discussed above, we tend to look for clues at viruses that are genetically similar. This may arise from the inability to obtain adequate information about a new virus. The proposed protein intrinsic disorder-based model can therefore serve this purpose by providing initial information that could be investigated into and be used to suggest new precautionary measures.
